# Single-Cell Dissection of the Immune Response After Acute Myocardial Infarction

**DOI:** 10.1161/CIRCGEN.123.004374

**Published:** 2024-05-16

**Authors:** Irene V. van Blokland, Roy Oelen, Hilde E. Groot, Jan Walter Benjamins, Kami Pekayvaz, Corinna Losert, Viktoria Knottenberg, Matthias Heinig, Leo Nicolai, Konstantin Stark, Pim van der Harst, Lude Franke, Monique G.P. van der Wijst

**Affiliations:** Department of Cardiology (I.V.B., H.E.G., J.W.B.), University Medical Center Groningen, Groningen, the Netherlands.; Department of Genetics (I.V.B., R.O., L.F., M.G.P.v.d.W.), University Medical Center Groningen, Groningen, the Netherlands.; Department of Cardiology, University Medical Center Utrecht, Utrecht, the Netherlands (P.v.d.H.).; Medizinische Klinik und Poliklinik I, University Hospital, Ludwig-Maximilian University, Munich, Germany (K.P., V.K., L.N., K.S.).; German Center for Cardiovascular Research, Munich Heart Alliance, Munich, Germany (K.P., V.K., L.N., K.S.).; Institute of Computational Biology, German Research Center for Environmental Health, Helmholtz Zentrum München, Neuherberg, Germany (C.L., M.H.).; Department of Computer Science, TUM School of Computation, Information & Technology, Garching, Germany (C.L., M.H.).; Department of Informatics, Ludwig-Maximilians Universität München, Munich, Germany (M.H.).

**Keywords:** coronary artery disease, immunity, single-cell gene expression analysis, ST-segment elevation myocardial infarction

## Abstract

**BACKGROUND:**

The immune system’s role in ST-segment–elevated myocardial infarction (STEMI) remains poorly characterized but is an important driver of recurrent cardiovascular events. While anti-inflammatory drugs show promise in reducing recurrence risk, their broad immune system impairment may induce severe side effects. To overcome these challenges, a nuanced understanding of the immune response to STEMI is needed.

**METHODS:**

For this, we compared peripheral blood mononuclear single-cell RNA-sequencing (scRNA-seq) and plasma protein expression over time (hospital admission, 24 hours, and 6–8 weeks post-STEMI) in 38 patients and 38 controls (95 995 diseased and 33 878 control peripheral blood mononuclear cells).

**RESULTS:**

Compared with controls, classical monocytes were increased and CD56^dim^ natural killer cells were decreased in patients with STEMI at admission and persisted until 24 hours post-STEMI. The largest gene expression changes were observed in monocytes, associating with changes in toll-like receptor, interferon, and interleukin signaling activity. Finally, a targeted cardiovascular biomarker panel revealed expression changes in 33/92 plasma proteins post-STEMI. Interestingly, interleukin-6R, MMP9 (matrix metalloproteinase-9), and LDLR (low-density lipoprotein receptor) were affected by coronary artery disease–associated genetic risk variation, disease status, and time post-STEMI, indicating the importance of considering these aspects when defining potential future therapies.

**CONCLUSIONS:**

Our analyses revealed the immunologic pathways disturbed by STEMI, specifying affected cell types and disease stages. Additionally, we provide insights into patients expected to benefit most from anti-inflammatory treatments by identifying the genetic variants and disease stage at which these variants affect the outcome of these (drug-targeted) pathways. These findings advance our knowledge of the immune response post-STEMI and provide guidance for future therapeutic studies.

**See Editorial by Yarahmadi & Nguyen**

ST-segment–elevated myocardial infarction (STEMI) is a major cause of mortality and morbidity. The immune system is crucial during both atherosclerotic plaque formation and rupture and in the consequent inflammatory response.^1,2^ Targeting this response has remained challenging due to its complexity and severe side effects, as seen in the CANTOS (Canakinumab Anti-inflammatory Thrombosis Outcome Study; anti-IL-1ß) and ASSAIL-MI (ASSessing the effect of Anti-IL-6 treatment in MI; anti-IL6R) trials.^3,4^ To overcome these challenges, the pathophysiological mechanisms post-STEMI should be studied in greater molecular and cellular detail.^5,6^

Previously, studying cell types relied on bulk analyses using predefined marker genes. Single-cell RNA-sequencing (scRNA-seq) now facilitates unbiased transcriptome-wide analysis of 100 000s of individual cells simultaneously.^7,8^ This technology reveals new insights into the inflammatory response post-STEMI by concurrently mapping changes in cell type composition, gene expression level, and downstream pathways at various cellular resolutions.

Both normal physiological processes in the heart^9^ and blood,^8^ and pathophysiological processes during atherosclerosis,^10,11^ and neovascularization,^12^ have been studied at the single-cell level. A spatial multi-omics map of the heart upon STEMI revealed an increased dependency between lymphoid and myeloid cells in ischemic samples compared with healthy controls, indicating their communication during cardiac repair.^13^ While many aspects of STEMI have been explored, a detailed single-cell view of how circulating immune cells are affected during acute and chronic phases remains lacking.

Here, we compared scRNA-seq data of 95 995 peripheral blood mononuclear cells (PBMCs) from 38 patients with STEMI (during hospital admission [t0]), 24 hours [t24h], and 6–8 weeks [t8w] post-STEMI) to 33 878 PBMCs from 38 age- and sex-balanced general population controls. This revealed large changes in cell type composition, gene and plasma protein expression levels, and cell-cell communication, both compared with controls and post-STEMI (Figure S1). Additionally, we found key plasma proteins affected by a combination of genetics, disease status, or disease phase. Altogether, this study presents the first single-cell view of the circulating immune system during STEMI and emphasizes the importance of considering person- and disease-related characteristics to fully grasp the underlying molecular changes.

## METHODS

Methods are available in the Supplemental Material. The ethics committee of the University Medical Center Groningen (Medical Ethical Review Committee-UMCG-2012/296) approved this study. All patients provided written, informed consent.

## RESULTS

### Patient Characteristics

**Figure 1.**
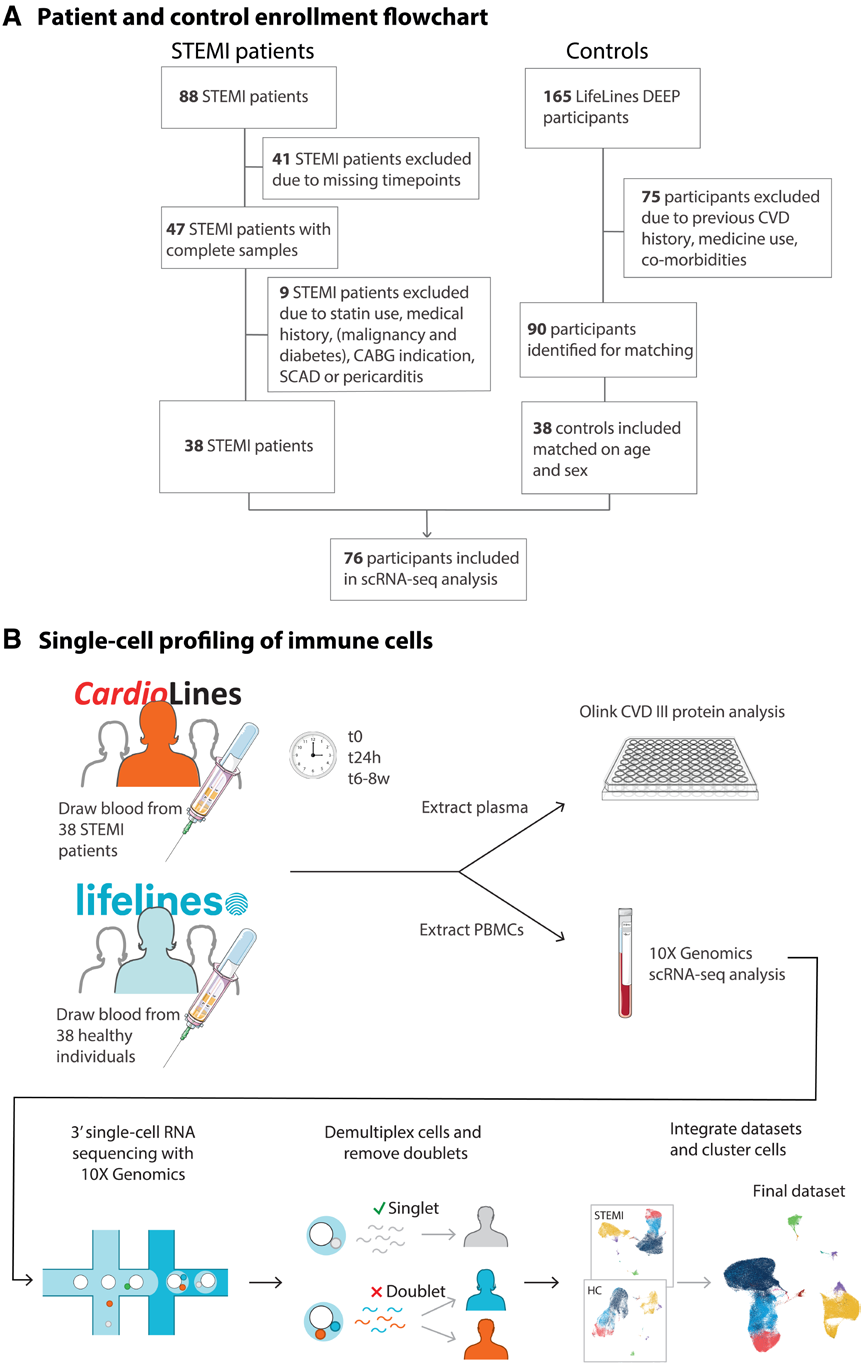
**Study overview. A**, Selection of patients with STEMI (CardioLines biobank) and controls (LifeLines DEEP biobank). **B**, Experimental setup. CABG indicates coronary artery bypass graft surgery; CVD, cardiovascular disease; HC, healthy control; PBMC, peripheral blood mononuclear cell; SCAD, coronary artery bypass graft surgery; scRNA-seq, single-cell RNA-sequencing; and STEMI, ST-segment–elevated myocardial infarction.

To dissect the immune response post-STEMI, PBMCs and plasma from 38 patients with a first STEMI were collected at t0, t24h, and t8w (Figure 1). Patients with STEMI were mostly male (84%), 60±11 years old, with a median body mass index of 26.4±3.5 kg/m^2^. Most patients presented with 1 stenosed vessel (55%), with 39% showing complete vessel occlusion (Table; Table S1). These patients were compared to previously published scRNA-seq data from 38 age- and sex-balanced controls in the LifeLines DEEP cohort.^7^

**Table.**
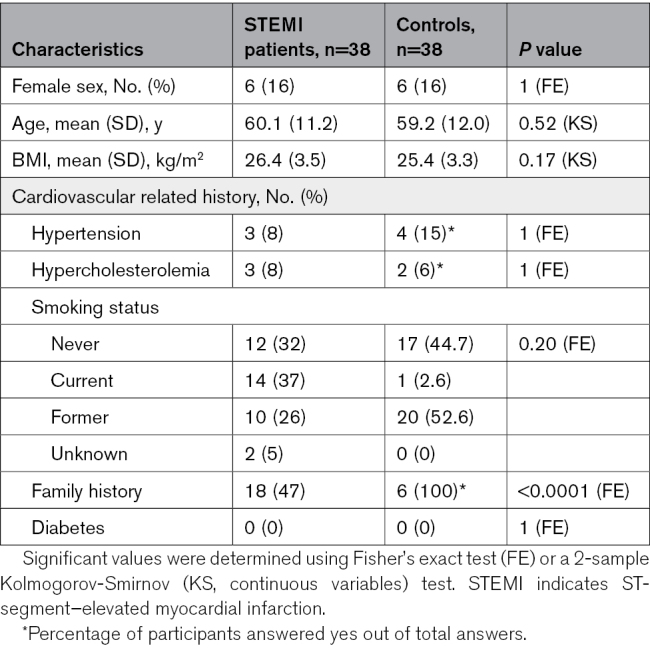
Baseline Characteristics

### Single-Cell Profiling of Immune Cells in Patients With STEMI and Controls

Collected PBMCs were used for 10X Genomics scRNA-seq analysis using v2 and v3 chemistries. We captured an average of 842 cells/individual/condition after quality control in patients with STEMI (v2: 831 genes/cell, v3: 1548 genes/cell) and 891 cells/individual in controls (v2: 1012 genes/cell, v3: 1931 genes/cell; Table S2). To annotate donors to cells and identify doublets, genotype-dependent demultiplexing was performed, revealing 1 incorrectly omitted donor-time point combination (t8w) and 10.0% doublets on average. Quality control was performed separately per chemistry due to technical differences. After quality control, 129 873 cells (95 995 diseased and 33 878 control) remained in the final data set. K-nearest neighbors clustering was performed on the normalized, integrated count data, allowing the identification of cell types.

### Monocytes and Natural Killer Cells Show Compositional Changes Post-STEMI

**Figure 2.**
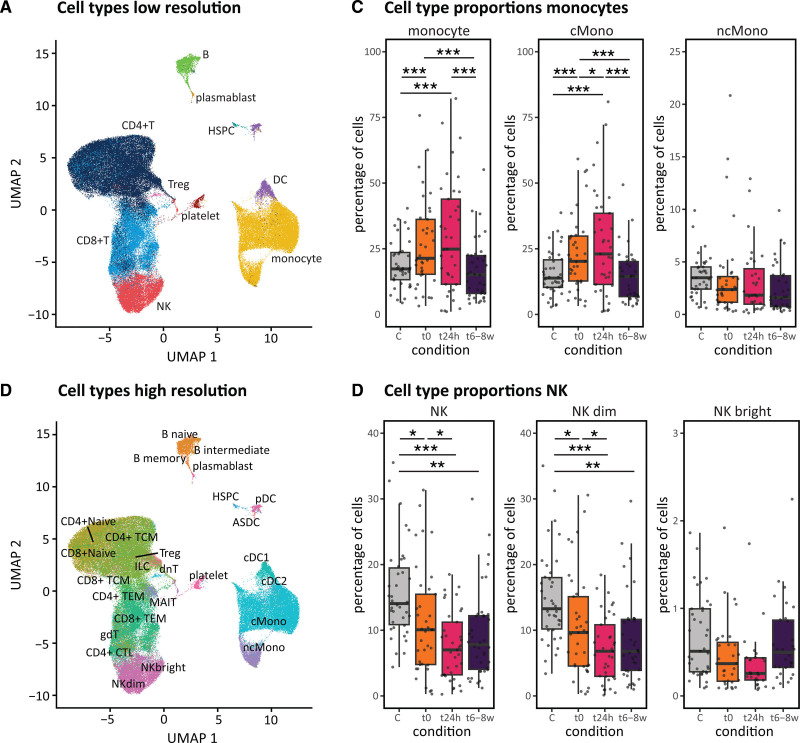
**Cell type composition changes.** Peripheral blood mononuclear cells (PBMCs) of patients with ST segment–elevated myocardial infarction (STEMI) (t0, t24h, and t6–8w) and controls presented in uniform manifold approximation and projections (UMAPs): **A**, showing the 10 major; **B**, 29 minor cell types. **C**, Proportions of monocytes (major class and its 2 subtypes) and (**D**) natural killer (NK) cells (major class and its 2 subtypes) in controls and post-STEMI. ASDC indicates AXL SIGLEC6 dendritic cell; C, control; cMono, classical monocytes; CTL, cytotoxic T cell; cDC, conventional dendritic cell; HSPC, hematopoietic stem and progenitor cell; ILC, innate lymphoid cell; MAIT, mucosal associated invariant T cell; ncMono, nonclassical monocytes; pDC, plasmacytoid dendritic cell; TCM, T-central memory; and TEM, T-effector memory. Significant Holm’s adjusted *P* value *P<0.05, **P<0.01, ***P<0.001. Each donor’s proportion depicts a cell count-weighted data point, each boxplot the weighted median, 25th and 75th percentile.

We identified 10 major cell types in the PBMCs of patients with STEMI and controls, including B, CD4T, CD8T, dendritic cell, hematopoietic stem and progenitor cell, monocyte, natural killer cell (NK), plasmablast, platelet, and other T cells (Figure 2A). These major cell types could be split into 29 minor cell populations (Figure 2B; Supplemental Methods).

To conduct a robust cell type composition analysis, we initially focused our analyses on the 6 most abundant cell types (Table S3), after which we zoomed in on the subtypes for the significant changes (Figure 2C and 2D). Compared with controls, monocytes from patients with STEMI during admission increased (Holm’s adjusted *P*=6.0×10^−5^) and NK cells decreased their relative abundance (Holm’s adjusted *P*=0.029; Figure 2C and 2D; Table S4). These changes primarily stemmed from compositional shifts within the classical monocytes (Holm’s adjusted *P*=6.0×10^−5^) and the NKdim (Holm’s adjusted *P*=0.040) subtypes (Figure 2C and 2D; Table S4). Similarly, during the disease course, we observed composition changes in monocytes (decrease t0–t8w: Holm’s adjusted *P*=0) and NK cells (decrease t0–t24h: Holm’s adjusted *P*=3.6×10^−2^) that stemmed from changes in classical monocytes (Holm’s adjusted *P*=8.0×10^−5^) and NKdim (Holm’s adjusted *P*=0.047; Figure 2C and 2D). Additionally, CD4T cells exhibited an increase (t0–t8w: Holm’s adjusted *P*=2.2×10^−2^; Figure S4) that could not be attributed to a specific cell subtype (Table S4). These analyses indicate the importance of analyzing compositional changes at higher resolution.

### Largest Gene Expression Changes Occur in Monocytes

**Figure 3.**
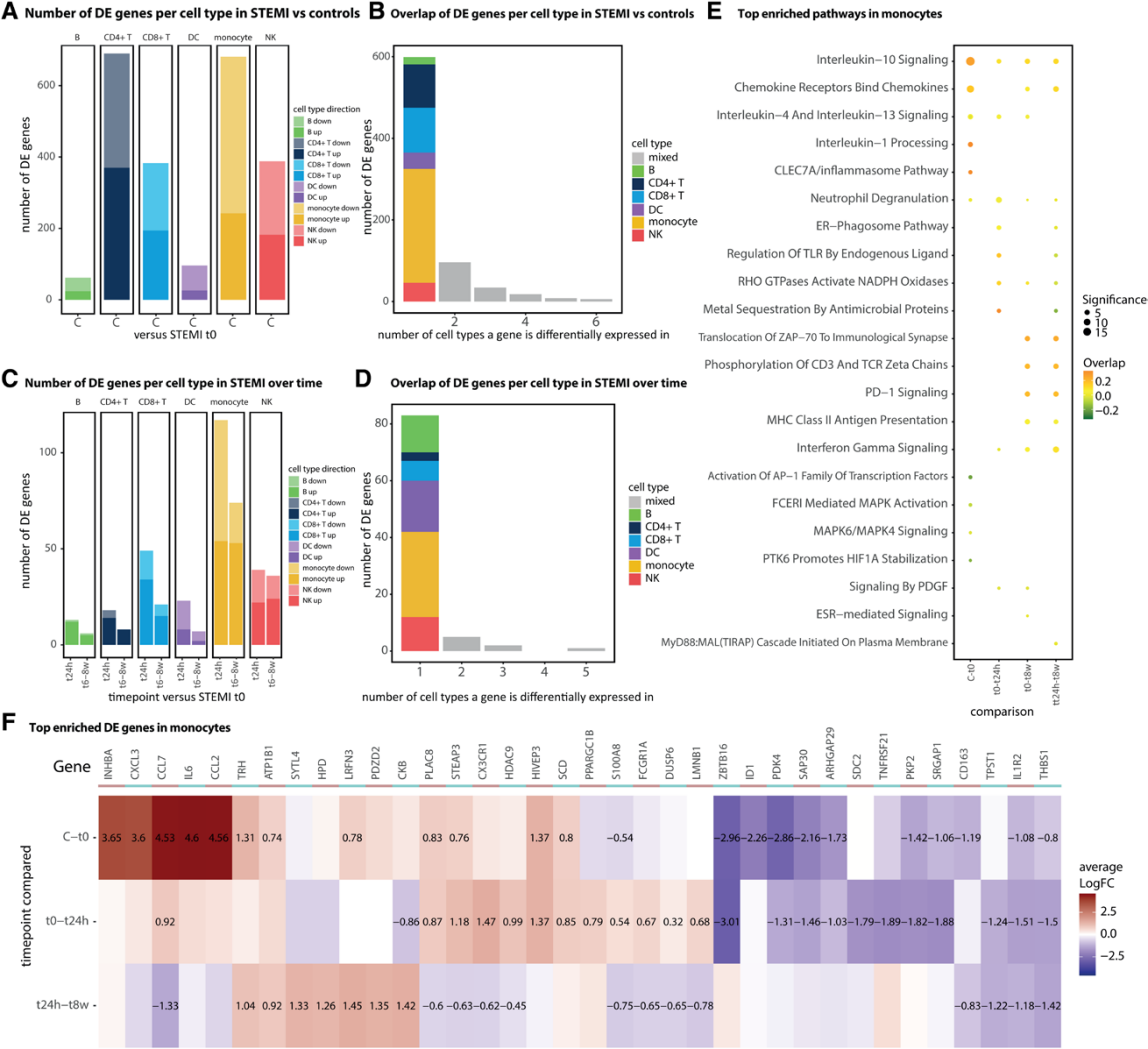
**Enriched differential expression (DE) genes and pathways. A**, The number of up- and downregulated DE genes/cell type at ST-segment–elevated myocardial infarction (STEMI) t0 (n=37) vs controls (n=38) or (**C**) in patients with STEMI over time (n=37 t24h, n=38 t8w). **B**, Overlap of DE genes in cell types at t0 vs controls or (**D**) in patients with STEMI over time (taking all DE genes significant in at least one of the comparisons [t0–t24h or t0–t8w]). **E**, Top enriched inflammatory pathways in monocytes between conditions. **F**, Heatmap showing the LFC of the maximum 10 most significant up and down DE genes in monocytes that are involved in the immune system pathway. Genes are hierarchically clustered and the LFC is only provided for significant differences. C indicates control; DC, dendritic cells; logFC, log fold change; and NK, natural killer cells.

To assign gene expression differences (DE) at STEMI t0 versus controls, we conducted pseudobulk DE analysis on the 6 major cell types using Limma Dream (Table S5). CD4T cells (690 genes) and monocytes (681 genes) showed the largest and B cells the least amount of change (62 genes; Figure 3A). Overall, more genes were downregulated (Figure 3A), and the majority were uniquely identified in monocytes (Figure 3B). We then split the DE genes into an up- or downregulated set and conducted pathway enrichment analysis separately (Table S6). The largest enriched pathway category was the immune response (Figure S6). Therefore, we focused on this category and observed that the strongest enrichment in monocytes was found among the upregulated DE genes; both pro- (IL-1) and anti-inflammatory (IL-4, IL-10, and IL-13) interleukins, chemokines, and the CLEC7A inflammasome pathway (acting upstream of IL-1) were enriched (Figure 3E and 3F).

Subsequent comparisons of DE post-STEMI revealed a general decrease in the number of DE genes from t24h to t8w (Figure 3C). Again, monocytes, but not CD4T cells, had the largest amount of total and unique DE genes post-STEMI (Figure 3C and 3D; Table S5). Similarly, these upregulated genes in monocytes were mostly enriched for immune response pathways (Figure 6S). During the disease course, the enrichment for anti-inflammatory pathways remained stable (IL-4 and IL-13) or further increased (IL-10), whereas genes involved in monocyte chemoattraction peaked at t24h (*CCL7*, *CX3CR1*; Figure 3E and 3F). Together with, but independently of, the observed cell type composition changes (Figure 2C and 2D), these results indicate that monocytes contribute significantly to the immunologic changes observed post-STEMI.

**Figure 4.**
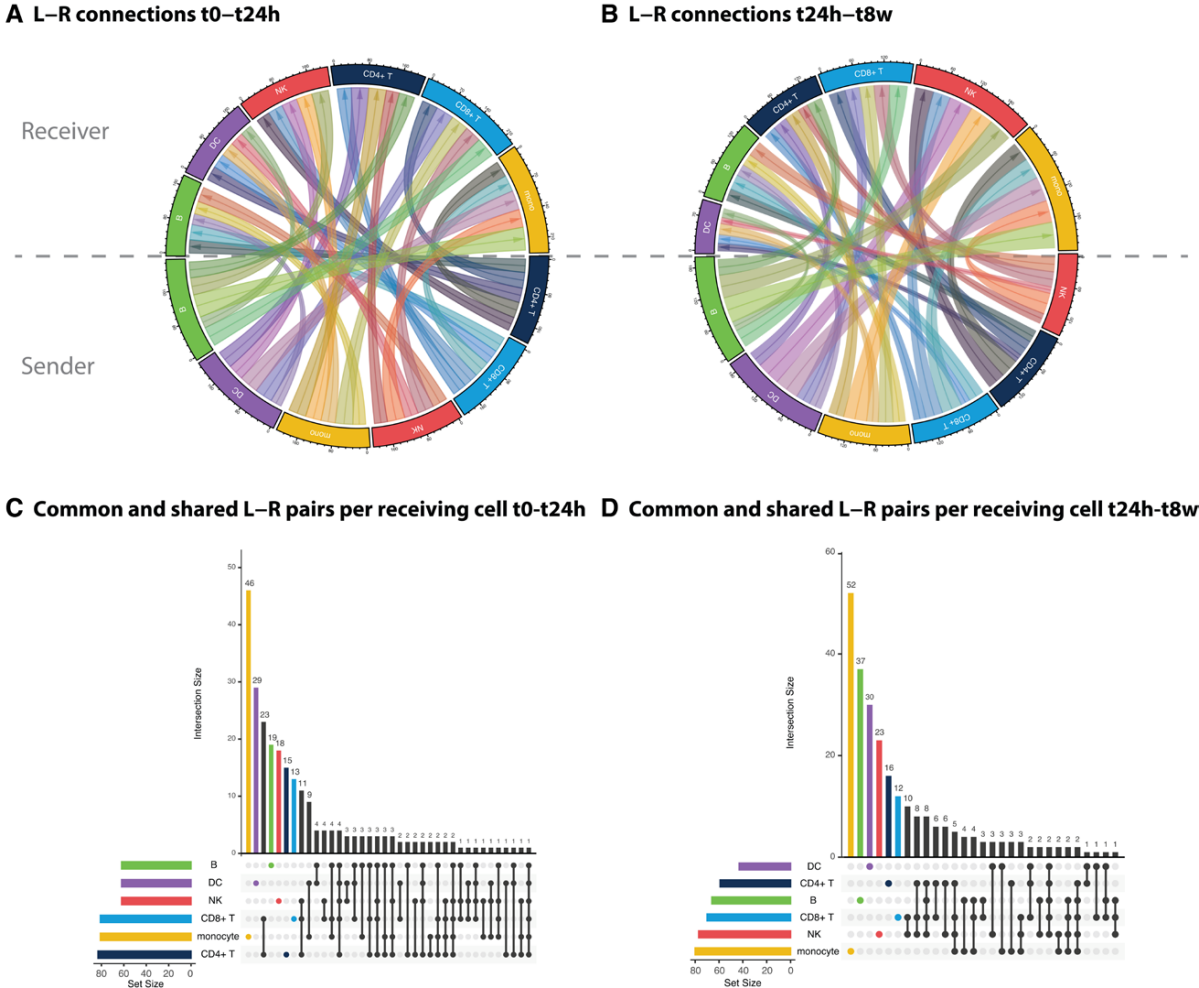
**Differential putative cell-to-cell communication post-ST-segment–elevated myocardial infarction (STEMI).** Differential incoming (receiver) and outgoing (sender) putative cell-to-cell communication at t0-t24h (**A**) and t24h-t8w (**B**). An active cell-to-cell communication link is counted as being a ligand-receptor (L-R) link that has resulted in differential downstream gene expression. For each active L-R link in **A** (t0–t24h) and **B** (t24h–t8w), the sharedness of ligands among the major cell types is depicted in **C** and **D**, respectively. The number of participants in each condition is 38, 37, and 38 for t0, t24h, and t8w, respectively. DC indicates dendritic cells; and NK, natural killer cells.

**Figure 5.**
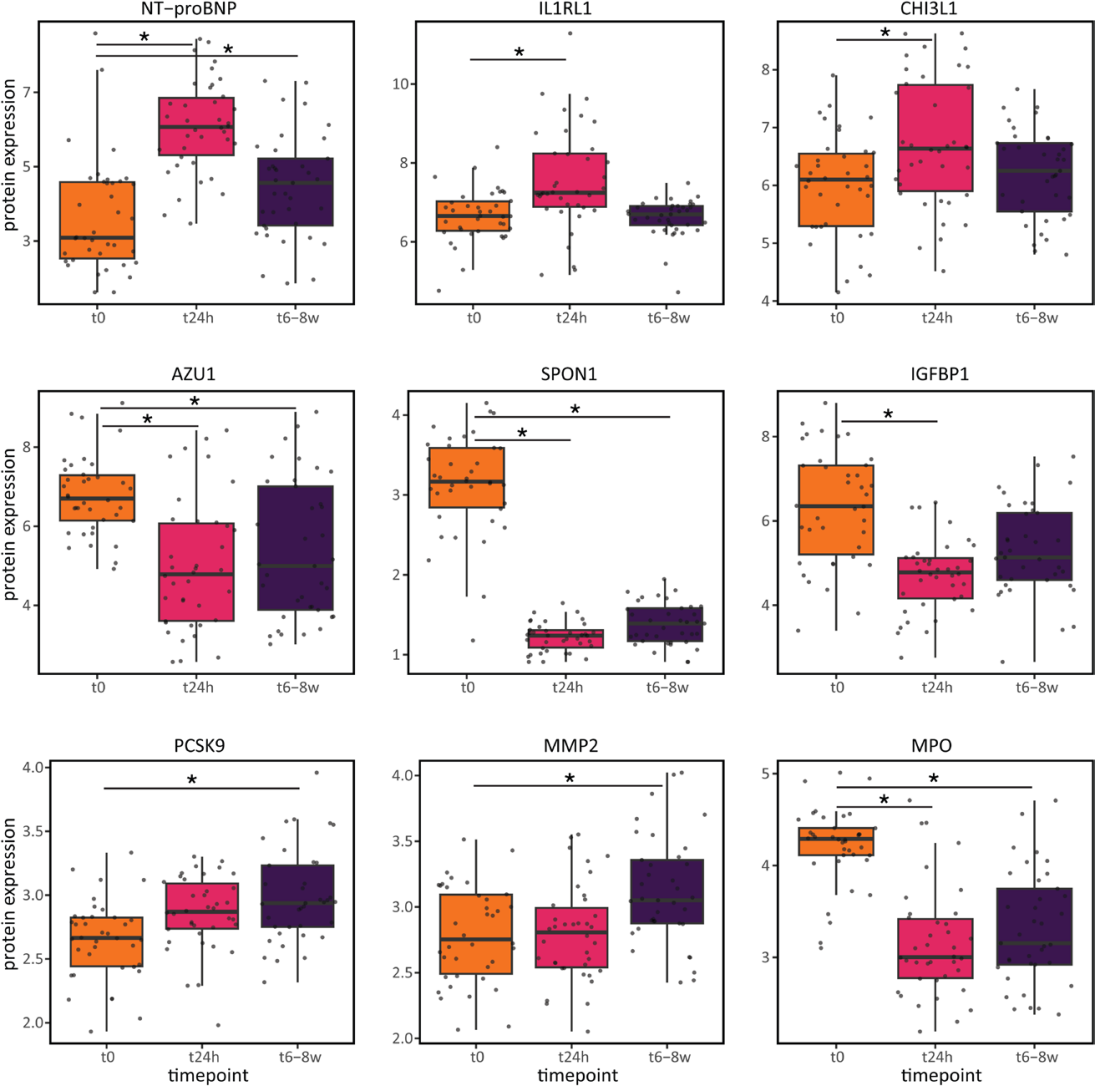
**Differentially expressed proteins post-ST-segment–elevated myocardial infarction (STEMI).** Top 3 up- and downregulated proteins at t24h (n=38) and t8w (n=37) post-STEMI vs t0 (n=38). *Significant Bonferroni-adjusted *P*<0.05. AZU1 indicates azurocidin1; CHI3L1, chitinase-3-like protein 1; IGFBP1, insulin-like growth factor-binding protein 1; IL1RL1, interleukin-1 receptor ligand 1; MMP2, matrix metallopeptidase 2; MPO, myeloperoxidase; NT-proBMP, N-terminal pro-B-type natriuretic peptide; PCSK9, proprotein convertase subtilisin/kexin type 9; and SPON1, spondin 1.

**Figure 6.**
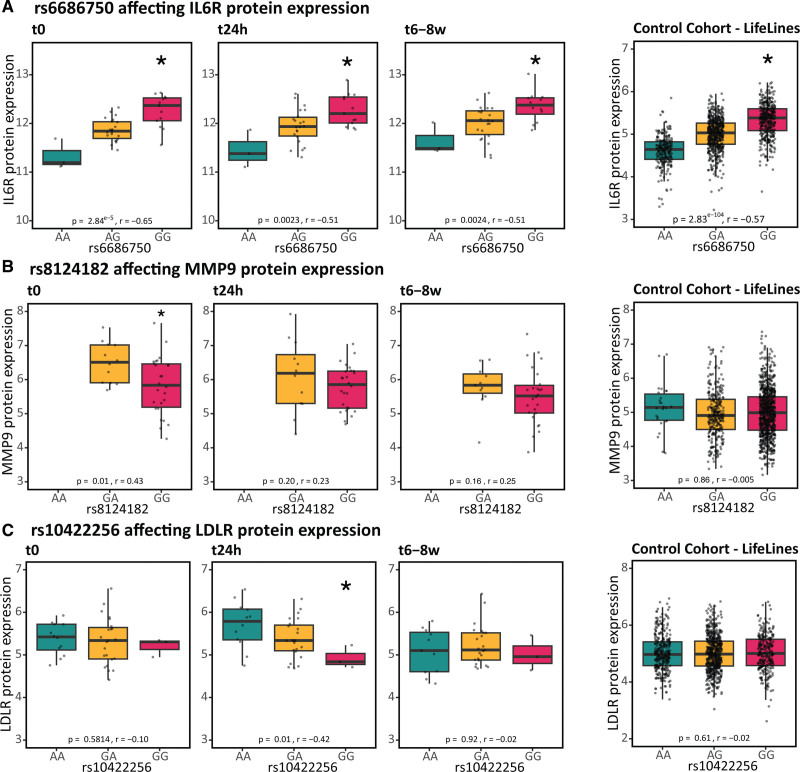
**Plasma expression/protein quantitative trait loci (pQTLs) in patients with ST-segment–elevated myocardial infarction (STEMI) vs controls show both condition-dependent and condition-independent changes.** Plasma pQTLs in 38 patients with STEMI and 1142 general population controls.^7^ In **A**, a pQTL is highlighted that is universal (i.e. found in both STEMI patients and the general population), whereas in **B** a pQTL is highlighted that is disease-specific (i.e. only in STEMI patients) or in **C** disease and condition-specific (i.e. only in STEMI patients 24h after hospital admission). The number of samples for each genotype group: Table S11. *FDR-corrected significant *P* values; r, Spearman correlation. IL indicates interleukin; LDLR, low-density lipoprotein receptor; and MMP9, matrix metalloproteinase 9.

### Monocytes Show Most Potential Differential Cell-to-Cell Communication Post-STEMI

As many of the enriched pathways were related to cell-to-cell communication (Figure 6S), we next defined how interactions among immune cell types could underlie these observed gene expression changes post-STEMI. For this, we used a cell-to-cell communication tool that identifies potential interactions by assuming that ligand-receptor interactions can be predicted based on ligand expression in 1 cell type and downstream gene expression changes of a known ligand-receptor interaction in another cell type (Table S7).^14^ In the acute phase (t0-t24h) of STEMI, the outcoming communication was largely balanced among the cell types, whereas the incoming communication was largest in the CD8T cells and monocytes (Figure 4A). In the chronic phase (t24h–t8w), the dendritic cells and B cells were the largest senders, and the NK cells and monocytes were the largest receivers (Figure 4B). Most of these ligand-receptor pairs were uniquely involved in communication by just 1 cell type; similar to the DE analyses, the monocytes showed the most unique interactions (Figure 4C and 4D). While this bioinformatically informed communication is solely a prediction and additional experimental follow-up would be needed for confirmation, the analyses have been conducted in a comparative fashion. Therefore, any method-derived biases will be comparable in each comparison and are not expected to affect the identified results.

### Cardiovascular Disease–Associated Proteins Change Post-STEMI

As changes in plasma proteins provide additional insights on the systemic consequences and are easily monitored in clinical follow-up, we then analyzed during the disease course the plasma levels of 92 proteins that are known or exploratory human cardiovascular and inflammatory markers. This revealed changes in 14 of 92 proteins during the first 24 hours (8 up and 6 down) and 28 of 92 proteins during the first 8 weeks (22 up and 6 down) post-STEMI. The top 3 upregulated proteins within the first 24 hours were NT-proBNP (N-terminal pro-B-type natriuretic peptide), IL1RL1, and CHI3L1 (Figure 5; Table S8). Both NT-proBNP and the soluble form of IL1RL1 are well-known proteins upregulated in response to increased wall stress during STEMI.^15^ Moreover, both are independent predictors of heart failure and cardiovascular death.^16,17^ CHI3L1 is an extracellular matrix protein involved in atherosclerosis and plaque rupture.^18^ This indicates that CHI3L1 upregulation could contribute to STEMI, whereas NT-proBNP and IL1RL1 reflect STEMI-induced mechanical stress on cardiomyocytes. The top 3 downregulated proteins during the acute phase were SPON1 (spondin-1), AZU1 (azurocidin 1), and IGFBP1 (insulin-like growth factor-binding protein 1). Each of these has been previously associated with processes preceding STEMI (eg, atherosclerosis for AZU1^16^ and IGFBP1^19^) or resulted from unsuccessful STEMI treatment (eg, worsened systolic heart function for SPON1^20^). Therefore, their sustained downregulation may indicate effective treatment, leading to restored blood flow and eventually preserving heart function.

During the chronic phase, the top 3 upregulated proteins were NT-proBNP, PCSK9 (proprotein convertase subtilisin/kexin type 9), and MMP2. The increased NT-proBNP levels may reflect further increased wall stress.^15^ PCSK9 is involved in degradation of the LDLR (low-density lipoprotein receptor)^21^ and might therefore reflect hyperlipidemia or patients’ statin treatment.^22^ MMP2 acts in the fibrotic pathway^23^ and is involved in cardiac remodeling.^24^ Both SPON1 and AZU1 continued to be downregulated at t8w. Additionally, MPO (myeoloperoxidase) was found to be downregulated, a protein contributing to plaque destabilization through local oxidative tissue injury.^25^ Together, these changes suggest a sustained restoration of heart function and blood flow.

When trying to assign the potential cell type responsible for these differential protein changes using scRNA-seq data, we could only replicate 4 of 34 DE proteins (75% concordant) in at least 1 cell type within the same condition comparison (Table S8). This low mRNA-protein replicability may reflect the inherent differences in what is captured: bulk plasma proteins (reflecting the secretome of PBMCS but also other blood cells or even other tissues) versus single-cell PBMC-derived mRNAs.

In a comparable cohort of 48 patients with STEMI, the same plasma proteins were compared at hospital admission and 3 months post-STEMI, showing 29 of 92 DE proteins.^26^ Twelve replicated in our study (100% concordant) and were mainly involved in STEMI-associated processes, like the immune response and tissue remodeling, indicating robustness (Table S8).

### Gene and Protein Expression Profiles Were Not Associated to Biochemical-Defined Infarct Size

Patients with STEMI exhibit variable symptoms upon presentation. Despite our study’s criteria narrowing clinical variation (Table), donor-to-donor variation persists, influenced by factors like age, sex, infarct size, and genetics. To assess the correlation between infarct size and molecular measurements, we used plasma peak CK-MB (creatine kinase myocardial band) levels as proxy.^27^ This biomarker reaches a peak within 24 hours and predicts left ventricle dysfunction. Neither cell type proportions nor monocyte gene expression showed a significant association with peak CK-MB at any time point. However, NT-proBNP was positively associated with peak CK-MB (t24h: r^2^-adjusted=0.49, Holm’s adjusted *P*=2.8×10^−3^; Table S9). This marker for myocardial wall stress is commonly measured to assess left ventricular dysfunction,^15^ and therefore, both are expected to be correlated during the time CK-MB peaks.^15,28^

### Coronary Artery Disease–Associated Genetic Risk Variants Show Disease- and Condition-Dependent Effects on Plasma Proteins of Known Drug Targets

Beyond infarct size, genetic variation contributes to a patients’ molecular response. Recognizing this is crucial when assessing a patient’s disease course and defining patient-tailored treatment. As plasma proteins provide a pool of potential therapeutic targets, we assessed the effect of genetic variation on these (pQTL [protein quantitative trait locus]). To provide a direct clinical link, we focused on coronary artery disease–associated variants.^29^ For 3 of 92 proteins, we detected a pQTL in at least 1 time point post-STEMI (Table S10). For these 3, we assessed their disease-specificity by comparing pQTL effect sizes with a control cohort of 1142 individuals from the general population. One pQTL was significant in all conditions and showed similar effect size in patients and controls: single-nucleotide polymorphism (SNP) rs6686750 affecting IL6R (patients with STEMI: genotype β=0.42, FDR-corrected *P*=1.6×10^−5^; Figure 6A). The 2 other pQTLs were disease- or even condition-specific: SNP rs8124182 affecting MMP9 was found specifically in patients with STEMI (genotype β=−0.68, *P*=0.016; Figure 6B) and SNP rs10422256 affecting LDLR was found only in patients at t24h (genotype*t24h interaction β=−0.29, FDR-corrected *P*=0.022; Figure 6C). These results indicate the importance of considering genetics when studying the relationship between STEMI and molecular phenotype.

## DISCUSSION

Here, we provided an unbiased, longitudinal overview of the single-cell immune response in patients with STEMI versus controls. We observed an important role for the classical monocytes, CD4T cells, and NKdim cells post-STEMI, both in terms of cell type compositional and gene expression changes. In circulation, we observed increases in the monocyte and decreases in the NK fraction that peak or dip, respectively, at 24 hours post-STEMI. These changes agree with previous studies in STEMI^30,31^ and angina pectoris.^30,32^ Furthermore, previous studies revealed biphasic changes in monocyte composition post-STEMI^30^; in the early ischemic phase (3 days post-STEMI), classical monocytes peaked, whereas later (5 days post-STEMI), the nonclassical fraction peaked. These changes in circulation co-occur with specific changes in the heart, suggesting an initial recruitment of classical monocytes to facilitate dead cardiac tissue removal, followed by nonclassical monocytes concluding the inflammatory response and promoting tissue repair.^33^ Altogether, the observed changes are not solely the consequence of the STEMI but may also contribute to it. Therefore, studying circulating immune cell and blood protein level changes during a STEMI may provide leads for novel therapies.

Our study indicated the importance of considering both genetic variation and disease phase when assessing the molecular consequences of a STEMI, as several of the plasma proteins were affected by a combination of these parameters. For all identified pQTLs, the A allele is the coronary artery disease–associated risk allele.^29^ Indicating that lower plasma protein levels of IL-6R (Figure 6A), and higher levels of MMP9 (Figure 6B) and LDLR (Figure 6C) increase coronary artery disease risk. This agrees with previous phenome-wide association studies showing that the risk allele of these pQTL is associated with lower IL6 and IL6R plasma levels and higher LDL cholesterol levels.^34^

We observed a disease-specific pQTL of rs8124182 on MMP9 (Figure 6B), aligning with a previously described whole blood *MMP9* eQTL.^35^ We hypothesize this disease-specificity is due to this SNP’s direct effect on phospholipid transfer protein (*PLTP*) gene expression,^35^ regulating MMP9 levels. Evidence from *Pltp/ApoE* double knock-out mice supports this, showing reduced MMP9 protein levels could be reversed by *Pltp* overexpression.^36^ Notably, this PLTP effect on MMP9 protein levels was observed solely in atherosclerotic conditions induced by ApoE knock out, explaining why the MMP9 pQTL was evident only in patients with STEMI, not in controls (Figure 6B).

We observed a disease-state- and disease-stage-specific (t24h) pQTL for LDLR. Normally, membrane-bound LDLR is mostly present in the liver, enabling LDL uptake from circulation. When ADAM17 sheds LDLR from the membrane into circulation, reduced availability of membrane-bound LDLR is expected, leading to decreased LDL uptake. ADAM17’s activity is stimulated by inflammation,^37^ which may explain why this pQTL on soluble LDLR manifests only during the acute inflammatory stage post-STEMI (Figure 6C).

Finally, our analyses shed new light on the IL-6 signaling pathway, which is currently being targeted by various drugs in clinical trials.^38,39^ IL-6 signaling can be activated through 3 signaling modes, each having its own consequences: classical signaling (anti-inflammatory), trans-signaling (proinflammatory), and trans-presentation (Th17-cell promoting).^38^ Through cell-cell communication analyses, we found differential activation of the classical and trans-presentation pathways post-STEMI. While our pQTL analysis indicated that trans-signaling was affected by SNP rs6689206 regulating soluble IL6R levels both in patients with STEMI and controls (Figure 6A). Together, this indicates that classical and trans-presentation signaling may be suitable targets (eg, anti-IL-6^38,39^ or anti-IL-6R) post-STEMI, while patients with the GG genotype at SNP rs6689206 may benefit from treatments targeting specifically the trans-signaling pathway (eg, soluble gp130).

Unfortunately, when focusing on how each of the molecular layers could be contributing to or being the consequence of biochemically measured infarct size, no such relationship was found except for plasma NT-proBNP levels (Table S9)—a known marker for heart failure.^15^ This lack of association might have resulted from assessing each molecular parameter separately or not considering all potentially relevant clinical and donor variables in the model.^40^ Such a model that considers all these parameters requires a larger sample size. We foresee future feasibility through meta-analyses across uniformly processed (disease) population-based single-cell data sets in efforts such as the single-cell eQTLGen consortium.^41^

In conclusion, this study highlights the importance of studying STEMI at a cell type–specific resolution while taking genetic variation, disease status, and disease phase into consideration. We expect that such an integrative approach will help to better grasp the molecular processes underlying STEMI and will be essential for the development of effective future therapies with reduced side effects.

## ARTICLE INFORMATION

### Acknowledgments

The images are created using Servier Medical Art and the authors are thankful to www.smart.servier.com for providing free online images.

### Sources of Funding

This study was supported by the Dutch Organisation for Scientific Research (NWO) Corona Fast-Track grant (440.20.001), an Oncode Senior Investigator grant, NWO VIDI grant (917.14.374). and NWO VICI grant (09150182010019) to Dr Franke. Dr van der Wijst is supported by a NWO VENI grant (192.029).

### Disclosures

None.

### Supplemental Material

Supplemental Methods

Figure S1–S6

Tables S1–S11

References 14,42–58

## Supplementary Material


