# Supplementary material for: Single-Cell Dissection of the Immune Response After Acute Myocardial Infarction
Source: Circ Genom Precis Med. 2024 May 16;17(3):e004374. doi: 10.1161/CIRCGEN.123.004374 (PMC11188632; doi:10.1161/CIRCGEN.123.004374)
Supplement: Supplementary file 1 [file hcg-17-e004374-s001.pdf]

Supplemental Material

Supplemental Methods

Study population

All patients presenting with a first STEMI upon admission at the Heart Catherization center at the University Medical Center Groningen (UMCG) were enrolled in the study between January 1st 2018 and November 30th 2019. Inclusion criteria were adults (>18 years) that had a STEMI, a primary percutaneous intervention (PCI) with implantation of at least one stent with a diameter of at least 3 mm resulting in thrombosis in myocardial infarction (TIMI) flow grade 2 or 3 post PCI, and which showed symptoms less than 6 hours before undergoing PCI (**Table S1**). Major exclusion criteria were previous myocardial infarction, medical history of diabetes, inflammatory disease or malignancies, medication affecting inflammation and clemastine or desloratadine use during intervention. Using these criteria, we initially enrolled 88 STEMI patients in this study, of which 41 were later excluded due to missing follow-up time points (t24h, t8w). These excluded patients were transferred to their regional hospitals within 24 hours of initial admission and therefore this timepoint was missed. Further 9 patients were excluded in the follow-up due to presence of exclusion criteria that were not yet known upon admission (**Figure 1**). Patient data was collected and managed using REDCap electronic data capture tools hosted at the UMCG.[42] The study was part of CardioLines, a single-center observational biobank aimed to study potential factors related to success or failure of diagnosis and treatment.[43] scRNA-seq data from 38 age- and sex-balanced participants from the LifeLines DEEP cohort were included as a control group.[7,44]

Clinical parameters

Upon inclusion of STEMI patients, whole blood samples were collected at three different timepoints: during admission at the heart catheterisation center (t0), 24 hours (t24h, acute phase) and 6-8 weeks (t8w, chronic phase) later (**Figure 1**). Standard laboratory assessment of the blood was performed upon admission and routine physical parameters were recorded (**Table 1, S1**). To determine the peak value, titres of creatine kinase (CK), myocardial band of CK (CK-MB) and troponin T were routinely measured at 3h, 6h, 9h, 12h, 24h and 48h after admission.

Isolation and preparation of PBMCs

PBMCs were isolated and stored as previously reported.[7] In short, for each donor and timepoint, whole blood was collected in two 10 ml EDTA-vacutainer (BD) tubes. Within 2 hours after collection, PBMCs were isolated from the blood using Vacutainer cell preparation tubes with sodium heparin. PBMCs were kept in RPMI1640 supplemented with 50 µg/mL gentamicin, 2 mM l-glutamine, and 1 mM pyruvate. Isolated PBMCs were cryopreserved in RPMI1640 containing 40% FCS and 10% DMSO. Within one year, PBMCs were further processed for scRNA-seq analysis. Cells were thawed in a 37°C water bath until almost completely thawed, after which the cells were washed with pre-warmed (37°C) RPMI1640. After washing, cells were resuspended in RPMI1640 medium and incubated for 1 h in a 45° slant rack at 37°C in a 5% CO_2 incubator. After resting, cells were washed twice in medium supplemented with 0.04% bovine serum albumin. Cells were counted using a hemocytometer and cell viability was assessed by the Trypan Blue assay (**Figure S2**).

Single-cell library preparation and sequencing

Fifteen sex- and timepoint-balanced sample pools were prepared aiming for 1,000 cells per patient for a total of 8 patients per pool. Single cells were captured with the 10X Chromium controller (10x Genomics) according to the manufacturer's instructions (document CG00026) and as described earlier.[45] Each sample pool was loaded into a different lane of a 10X chip (Single Cell A Chip Kit, PN-120236 v2 and PN-1000074 v3), divided over 15 10X runs in total. cDNA libraries for v2 and v3 were generated with the Single Cell 3' Library & Gel Bead kit (v2: 120237, v3: 1000075) and i7 Multiplex kit (120262) according to the company's guidelines. These libraries were sequenced using a custom program (28-8-150 (v3) vs 27-9-150 (v2)) on an Illumina NovaSeq6000 using a 150-bp paired-end kit.

Alignment and initial processing of sequencing data

CellRanger v3.0.2 software with default settings was used to demultiplex the sequencing data, generate FASTQ files, align the sequencing reads to the hg19 reference genome, filter cell and UMI (unique molecular identifier) barcodes, and count gene expression per cell. Genotypes of the samples were generated using the Infinium Global Screen Array-24 kit (v1 for the samples processed with 10X v2 chemistry and v3 for the samples processed with 10X v3 chemistry) and were phased using Eagle v2.4 and imputed with the HRC 1000G Phase-3 v5 (hg19) reference panel using the Michigan Imputation Server (Minimac v3).[46] Genotype information of the controls was processed as described in the Lifelines DEEP cohort paper.[44] Doublet detection and sample assignment were done using Souporcell,[47] confining ourselves to only use exonic single nucleotide polymorphisms (SNPs) with a MAF≥0.05. The detected doublets were discarded. The exact algorithm used in Souporcell, can be found in the original publication. In short, variant calling is performed on the 3'-ends of the RNA transcripts captured for each cell (10X barcode). This builds a genetic profile for each individual cell. The genotypes of these cells are then clustered, which will assign each cell to a cluster. Cells located between clusters will be called as doublets. The souporcell pipeline was run for each multiplexed 10x experiment separately. Then, samples were assigned to the cell cluster with the highest correlation between the genotype retrieved from the cell cluster versus the genotypes from the individuals that had been pooled in the same 10x experiment.

Data preparation and quality control

Version 4.0 of the R package Seurat was used for data preprocessing.[48,49] The data was split into four subsets: The cells from controls that were processed using 1. 10x version chemistry 2 (Cv2) or 2. version chemistry 3 (Cv3), or the cells from the STEMI cohort that were processed using 3. 10x version chemistry 2 (STEMIv2) or 4. version chemistry 3 (STEMIv3). The SCTransform method[50] was ran separately on each of these subsets, fitting a negative binomial model, and saving the residuals. Raw counts were then corrected using the residuals. PCA was then calculated on the corrected count matrices. The four subsets were integrated using the Canonical Correlation Analysis (CCA) method in Seurat: Shared correlation structures were identified using the first 30 principal components between datasets. These vectors were then aligned using dynamic time warping. The first 30 principal components were calculated on the integrated dataset and were used for cell clustering using Seurat's FindNeighbors (dims = 1:30) and FindCluster function (default parameters, resolution 1.2) and a UMAP plot was used to visualize this. All genes that were not detected

in at least 3 cells were removed. Cells with a high percentage of reads mapping to mitochondrially-encoded genes (>8% for 10X V2 and >15% for 10X V3) were discarded, as this can be a marker of poor-quality cells.[51] Also, cells expressing ≤200 genes were considered outliers and discarded. Furthermore, cells expressing ≥10 UMIs of the HBB gene were discarded, indicative of erythrocytes. Finally, all cells that were marked as doublets or inconclusive by the souporcell method were discarded.[47] In total, 129,873 cells were used for downstream analysis consisting of 95,995 cells from STEMI patients and 33,878 cells from age- and sex-balanced controls (**Table S2**).

Cell Type Classification
Cells were annotated in each dataset separately using Seurat's Azimuth method by projecting them on a previously annotated multimodal CITE-seq (combined scRNA-seq and protein expression) reference dataset of 162,000 PBMCs.[52] These annotations were transferred to the SCTransform integrated object. Each cluster was assigned the cell type that was the most prevalent guided Azimuth annotation in that cluster. Annotations were confirmed using marker gene expression (**Figure S3**). As a result of discordant marker gene expression, two clusters were reassigned: cluster 25 from ncMono to cMono and cluster 17 from doublets to ncMono. Major and minor cell types were defined as T cells (regulatory T cells (Treg), CD4T+ and CD8+ T-naive, CD4T+ and CD8T+ T central memory (TCM), CD4T+ and CD8+ T effector memory (TEM), CD4T+ cytotoxic (CTL), CD4T+ and CD8T+ proliferating, Mucosal mucosal associated invariant T cell (MAIT), double negative T (dnT), gamma-delta T (gdT), innate lymphoid cells (ILC), B cells (B naive, B intermediate, B memory), NK cells (NK dim, NK bright, NK Proliferating), monocytes (classical (cMono) and non-classical monocytes (ncMono)) and dendritic cells (AXL+ SIGLEC+ Dendritic Cell (ASDC), conventional DC1 (cDC1), cDC2, plasmacytoid DC (pDC)).

Cell type abundance
Cell type proportion differences across STEMI timepoints and differences between STEMI patients and controls were determined using the permutation-based method described in Farbehi *et al*.[53] The number of cells of each major cell type were summed across controls or patients (separately for each STEMI timepoint). Pairwise comparisons were made across all combinations of STEMI timepoints and the controls using a W-value of 0.05. Resulting p-values were Holm-Bonferroni corrected, with a corrected p-value < 0.05 being considered statistically significant.

Differential gene expression and pathway analysis
For DE analysis library-size normalization was performed by scaling the total UMI gene expression of each cell barcode to 10,000, after which the gene expression data was log-transformed (LogNormalize in Seurat). For each cell type, pairwise DE analysis was performed using Limma-DREAM[54] comparing everything to t0 (controls, 24 hours and 6-8 weeks), controlling for donor, disease condition/stage, age, batch and 10x version chemistry that the sample was processed with: $gene\ expression \sim condition + age + version\ chemistry + (1|\ donor)$. To correct for multiple testing, Bonferroni correction was applied and adjusted p-values <0.05 were considered statistically significant. The up- and downregulated DE genes were used for separate pathway analyses using the Enrichr functional enrichment tool, selecting for the REACTOME database to identify relevant pathways.[55] Details on Enrichr can be found in the original publication. In short, a Fisher-

exact test is performed to determine if gene sets are overrepresented when compared to their gene frequency expected by chance. The p-values of these gene sets are then corrected using Benjami-Hochberg. We considered corrected values of <0.05 as significant.

Cell-to-cell communication analysis

We predicted potential cell-to-cell interactions using NicheNet analysis v1.1.0.[56] Using a background set of DE genes, NicheNet predicts potential ligand-receptor interactions and predicts whether specific cell-cell interactions led to differences in downstream gene-expression. To determine DE ligands and receptors, as well as genes downstream of cells potentially interacting with the ligands, Limma Dream[54] was used on gene expression pseudobulked per gene per donor per cell type and condition:
$gene\ expression \sim disease\ stage + age +\ version\ chemistry + (1|\ donor)$.
Differential ligand and downstream expression changes were confined to genes that were present in at least 10% of the cells. The absolute log fold change between the conditions needed to be at least 0.1 to be considered for testing. We assessed potential ligand-receptor interactions between pairs found in the Omnipath database.[57] We then used NicheNet to assess ligand-receptor activity, by filtering predicted ligand-receptor interactions for those ligands that were shown to influence downstream gene expression.

Protein Olink analysis

Plasma from the 38 STEMI patients was collected at each of the three timepoints (t0, t24h, t8w), but missing one sample at t8w (n=37), and stored at -80°C. Plasma samples were analyzed for 92 cardiovascular disease related protein biomarkers by Olink Proteomics (Olink Target 96 Cardiovascular-III panel, Uppsala Sweden). The data received was QCed by excluding all proteins with a missing data frequency of >15% and samples with an Olink warning QC outcome from analysis. All samples with a low detection rate were given the limit of protein detection value for the specific protein. Using these QC parameters, none of the proteins had to be excluded.

Relating clinical variables to cell type abundance, gene and protein expression

Cell type proportions and gene and protein expression profiles were associated with peak CK-MB values in STEMI patients. For gene expression associations we used MAST[58], a two-part generalized linear model on the log-transformed count matrix, and correcting for age, sex, 10X chemistry and 10X lane. For cell type proportion and protein expression associations, we first conducted a centered-log-ratio or log-transformation, respectively, after which linear regression was conducted, correcting for sex and age.

pQTL analysis

For STEMI patients individual Olink plasma protein levels were linked to specific genetic variants (pQTL mapping) at each of the 3 timepoints (t0, t24h, t8w) using the eQTL mapping pipeline (https://github.com/molgenis/systemsgenetics/wiki/QTL-mapping-pipeline). We limited ourselves to the 241 variants that were previously found to show a genome-wide significant GWAS signal for CAD.[29] pQTLs were mapped per time point, only testing the SNPs within a 100kb window of the transcription start site of the gene transcribing the assessed protein. The significant pQTLs in STEMI patients in at least one timepoint were then assessed in controls by mapping these pQTLs in 1142 individuals from the general population cohort Lifelines Deep, in which the same Cardiovascular Olink panel was

previously measured[59] and genotype data was available. Genetic variants were converted to Trityper format using GenotypeHarmonizer (https://github.com/molgenis/systemsgenetics). To assess whether the pQTL effect strength was influenced by the timepoint after the STEMI (t0, t24h, t8w), we then conducted an interaction analysis by fitting a generalized linear model correcting for age and sex as fixed effects, and the participant as a random effect.

Statistical analysis

Discrete variables were represented as frequencies and percentages. Continuous variables with a normal distribution were summarized as mean ± standard deviation and if skewed were represented as median with interquartile range. Spearman correlation was used for non-normally distributed variables. Differences in protein levels were calculated using the Wilcoxon signed rank test. For DE gene and protein analysis, and their correlation to peak CK-MB values, multiple testing correction was performed using Holm-Bonferroni. We considered a corrected P-value <0.05 statistically significant. Statistical analysis and the figures were created using R Core Team (2020).

Data availability

Processed (de-anonymized) scRNA-seq data, including a text file that links each cell barcode to its respective individual, has been deposited at the European Genome-Phenome Archive (EGA), which is hosted by the EBI and the CRG, under accession number EGAD00001010064. Raw and processed gene expression count matrices, and metadata can be found on the website accompanying our paper: https://eqtlgen.org/sc/datasets/blokland2024-dataset.html.

Code availability

The code for Seurat v4 (https://github.com/satijalab/seurat), Eagle v2.x (https://github.com/poruloh/Eagle), Souporcell v1.x (https://github.com/wheaton5/souporcell) and our in-house eQTL pipeline2 v1.4.0 (https://github.com/molgenis/systemsgenetics/tree/master/eqtl-mapping-pipeline) can be found at GitHub. All custom code is also available via GitHub (https://github.com/molgenis/STEMI-scRNA-seq).

Supplementary Tables

Table S1**:** Extended baseline characteristics of STEMI patients
Table S2: QC and preprocessing
Table S3: Absolute cell counts per donor, split by major cell type and STEMI timepoint
Table S4: eLIFE output DPA (major and minor cell types)
Table S5: DE mRNA analysis
Table S6: Pathway analysis, pathway upregulated in condition 1 compared 2
Table S7: Cell-cell communication output of L-R and L-T interactions
Table S8: Differential protein analysis
Table S9: peak CK-MB by protein expression
Table S10: pQTL summary stats
Table S11: pQTL sample sizes

Supplementary figures

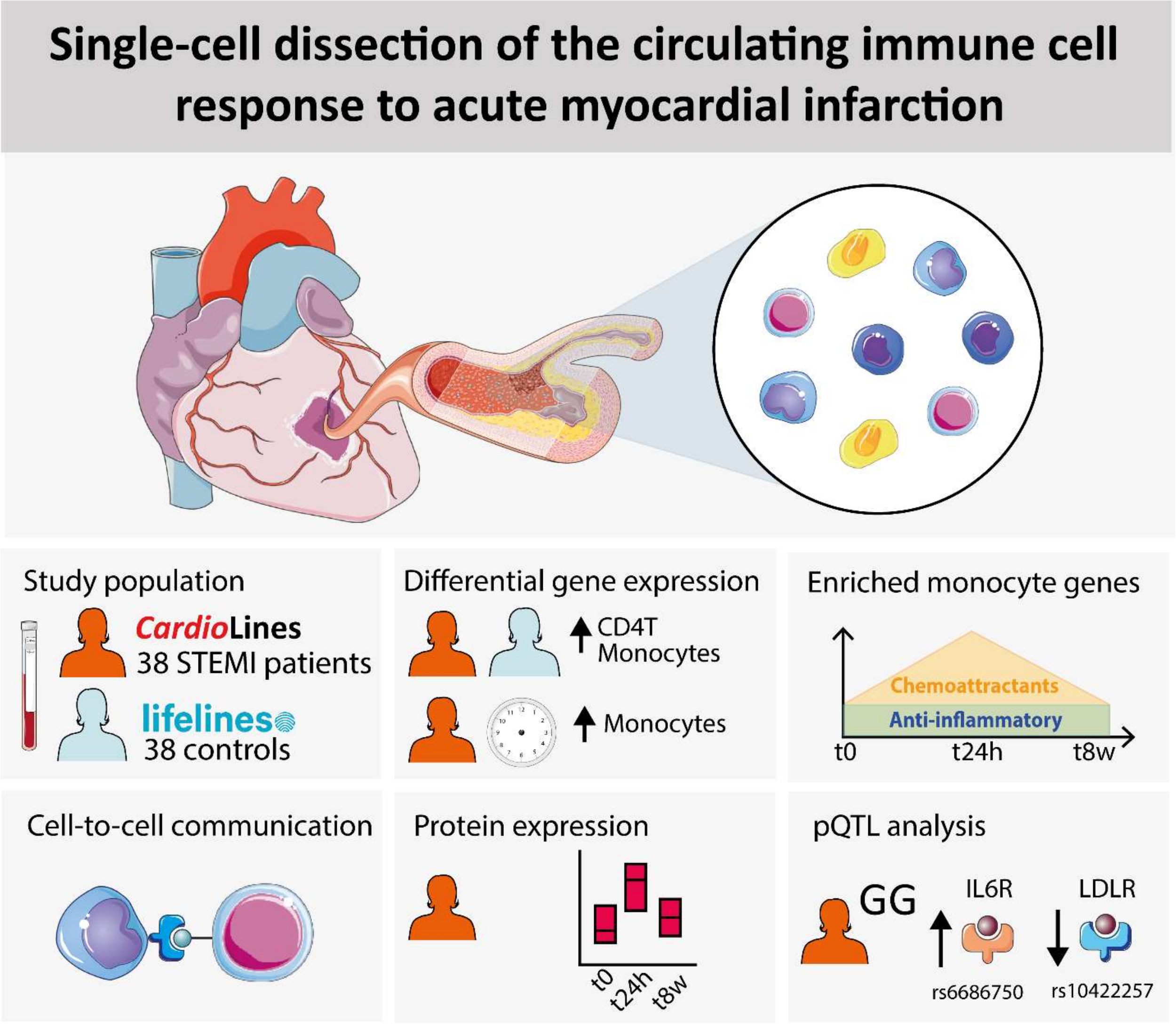

Figure S1*. *Study overview. *pQTL = protein quantitative trait locus; STEMI = ST-elevated myocardial infarction.*

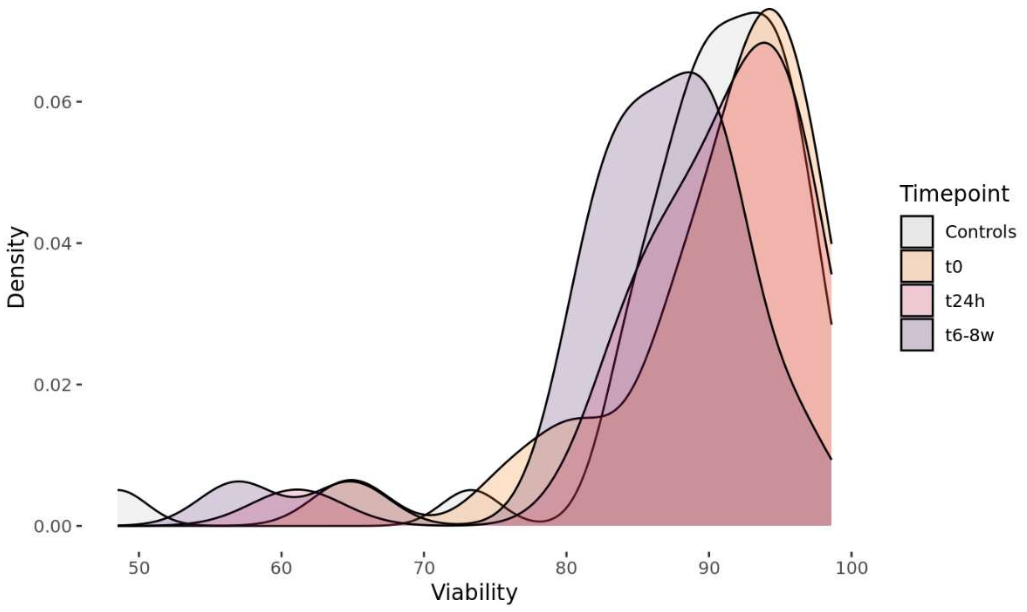

Figure S2. Cell viability per condition. *Histogram showing the PBMC cell viability as measured by Trypan Blue in STEMI patients over time (t0, t24h, t6-8w) and controls for cells prior to loading for scRNA-seq.*

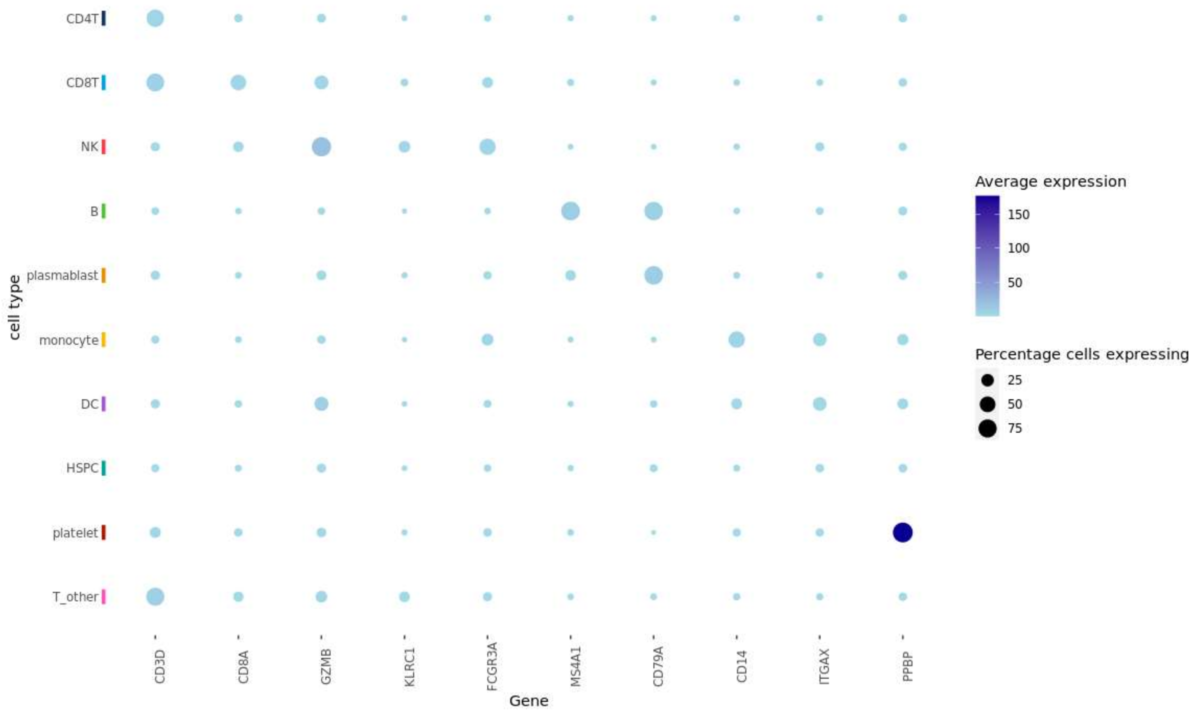

Figure S3*. *Marker gene expression. *Dotplot showing marker gene expression per cell type. Each cluster was assigned the most common Azimuth-annotated cell type. Correct assignment was validated using marker genes. DC = dendritic cells; HSPC = hematopoietic stem cells; NK = natural killer cells.*

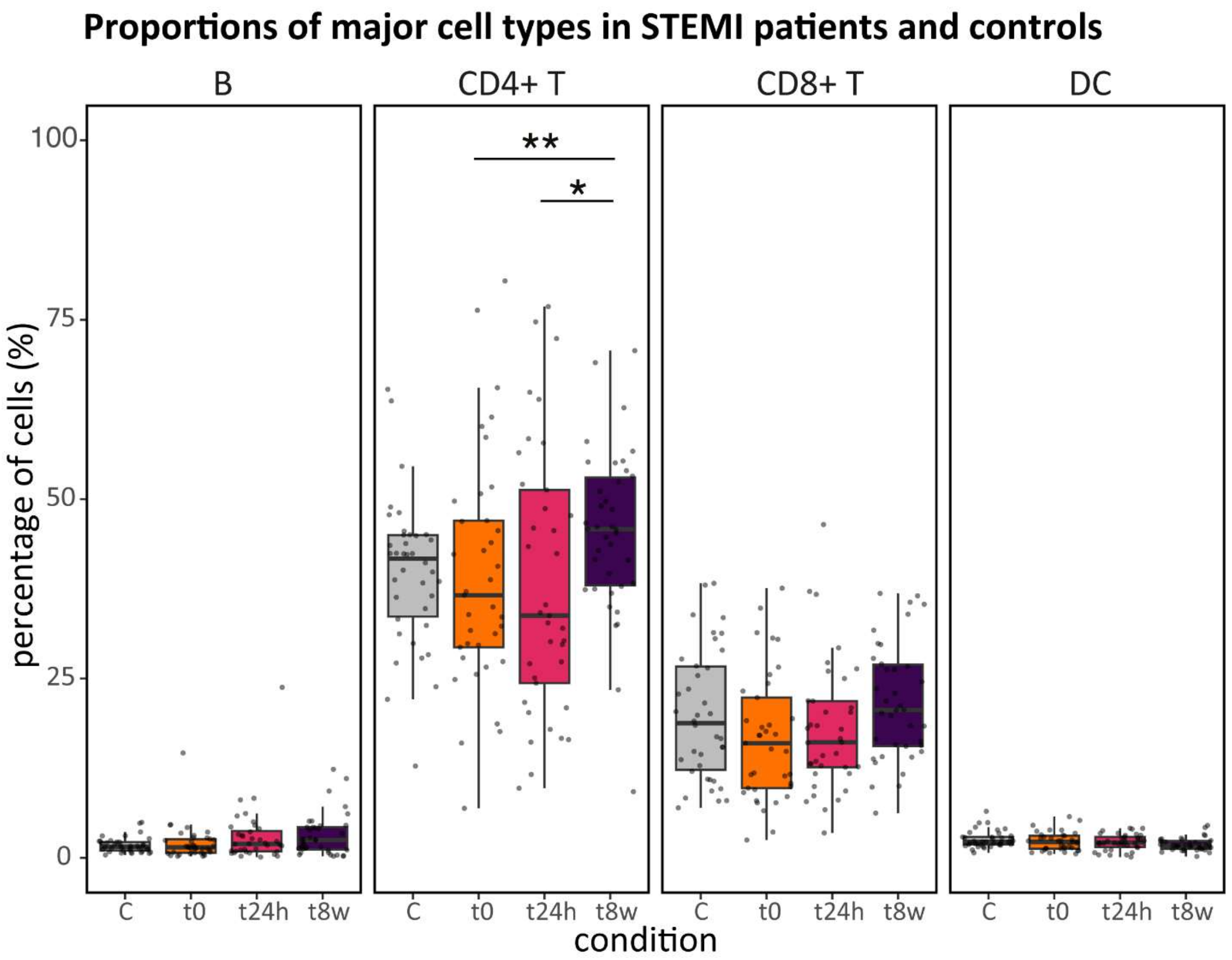

Figure S4. ***Cell type proportions of major cell types***. *Cell proportions in STEMI patients versus controls (C) and over time at three different time points (t=0, t=24 hours and t=6-8 weeks post STEMI). Significant differences are denoted as Holm´s-adjusted p-value *p<0.05, **p<0.01, ***p<0.001. n is 37, 37, 38 and 38 for t0, t24h, t8w and C respectively.*

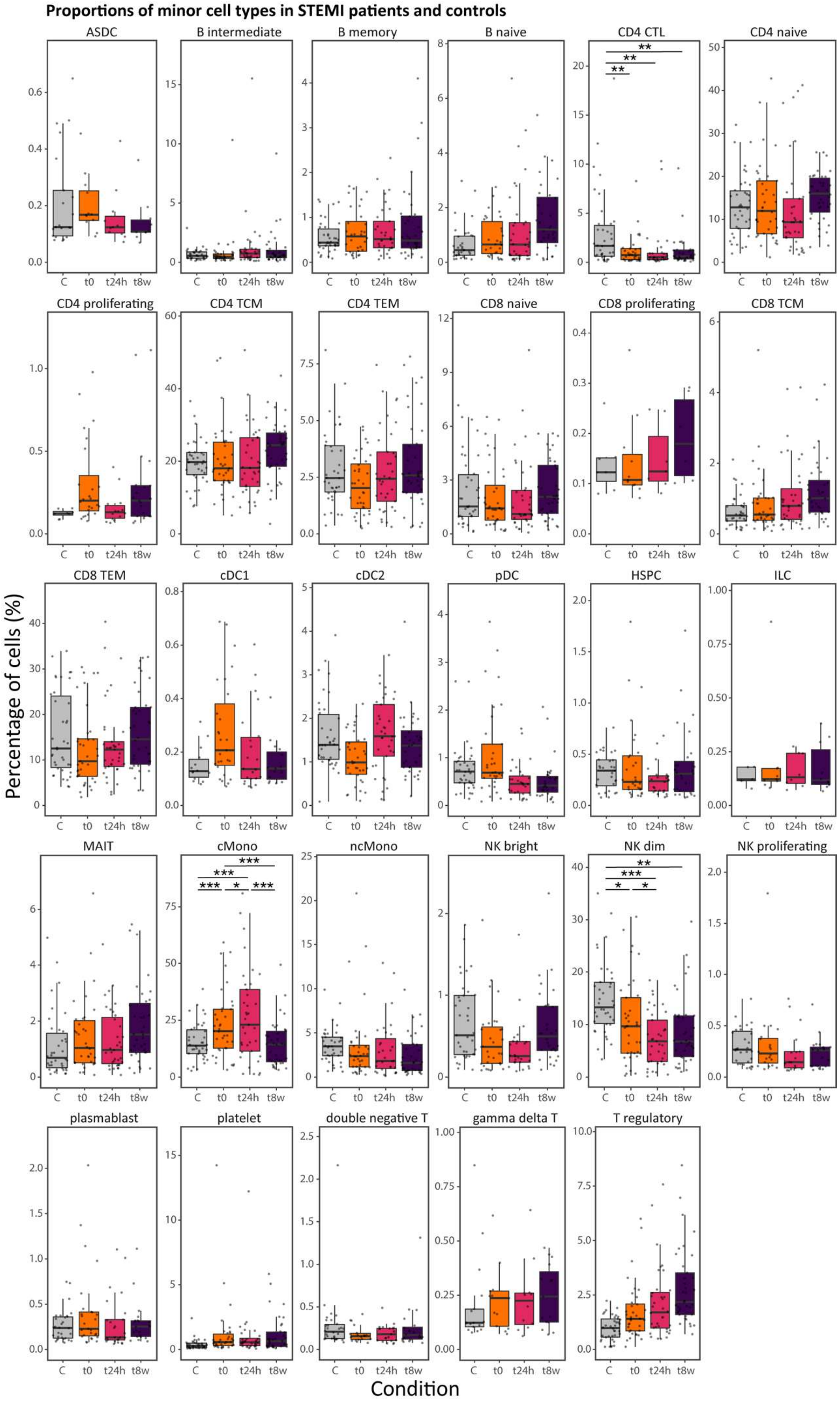
Proportions of minor cell types in STEMI patients and controls
ASDC
B intermediate
B memory
B naive
CD4 CTL
CD4 naive
CD4 proliferating
CD4 TCM
CD4 TEM
CD8 naive
CD8 proliferating
CD8 TCM
CD8 TEM
cDC1
cDC2
pDC
HSPC
ILC
MAIT
cMono
ncMono
NK bright
NK dim
NK proliferating
plasmablast
platelet
double negative T
gamma delta T
T regulatory
C t0 t24h t8w
Percentage of cells (%)
Condition

Figure S5. Cell type proportions of minor cell types*. Cell proportions in STEMI patients versus controls (C) and over time at three different time points (t=0, t=24 hours and t=6-8 weeks). Each donor's proportion is plotted as a datapoint. Each datapoint is then weighted by cell count and the weighted median, 25th and 75th percentile are plotted in a boxplot. ASDC = AXL SIGLEC6 dendritic cell; cMono = classical monocytes; CTL = cytotoxic T cell; cDC = conventional dendritic cell; HSPC = hematopoietic stem and progenitor cell; ILC = Innate lymphoid cell; MAIT = mucosal associated invariant T cell; ncMono = non-classical monocytes; pDC = plasmacytoid dendritic cell; TCM = T-central memory; TEM = T-effector memory. Significant differences are denoted by Holm´s-adjusted p-value as *p<0.05, **p<0.01, ***p<0.001. n is 37, 37, 38 and 38 for t0, t24h, t8w and C respectively.*

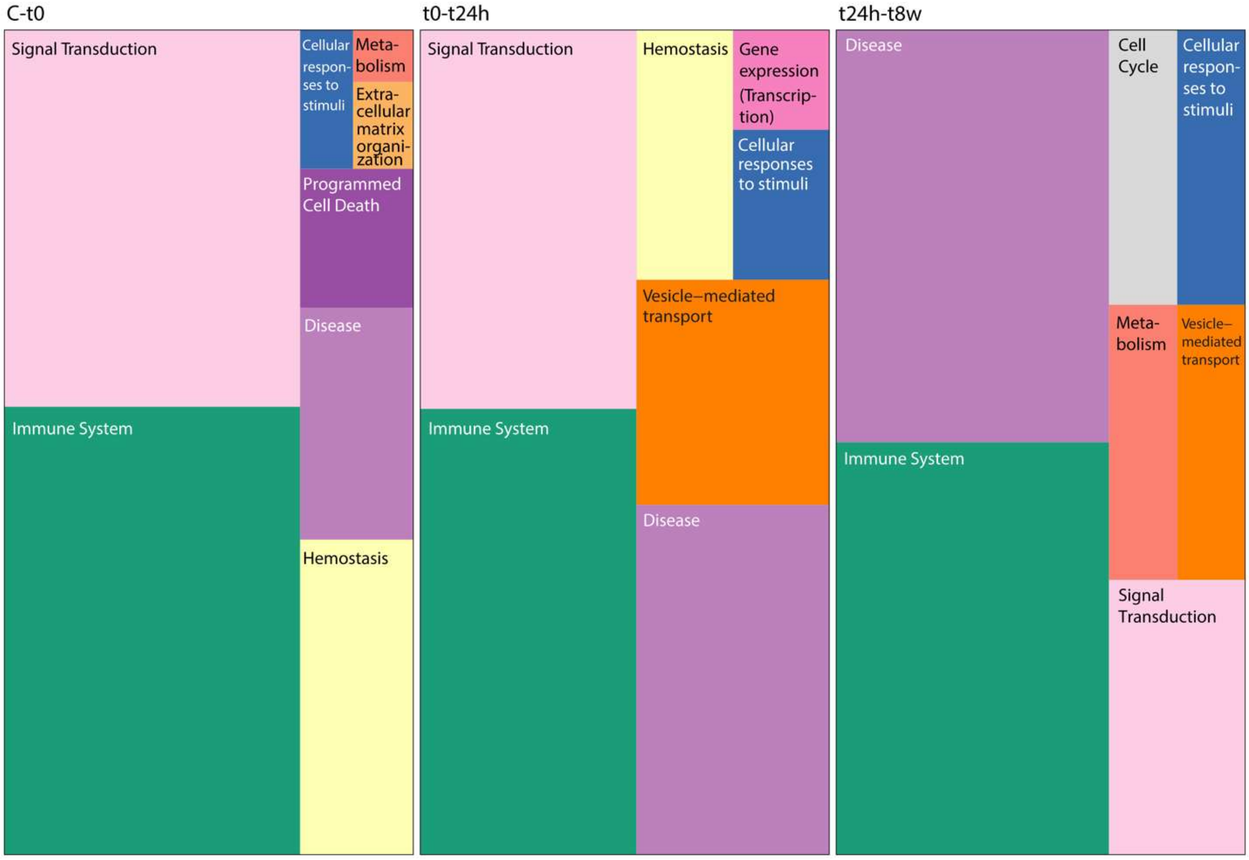

Figure S6*. *Pathway enrichment category. *Treeplot depicting the parent pathways that are enriched among the upregulated DE genes in monocytes in C vs t0, t0 vs t24h and t24 vs t8w. The area per category depicts the number of DE genes that are enriched in a specific parent pathway out of the total number of genes associated with that pathway.*
